# Association of placental manganese levels, maternal gut microbiota, and preeclampsia: a tripartite perspective

**DOI:** 10.3389/fmicb.2025.1674549

**Published:** 2025-10-20

**Authors:** Tianze Ding, Xiaoli Huang, Shiwei Ai, Yudong Pu, Wenting Zhao, Shuzhen He, Yuhui Dang

**Affiliations:** ^1^Institute of Maternal, Child and Adolescent Health, School of Public Health, Lanzhou University, Lanzhou, China; ^2^The Affiliated Dongguan Songshan Lake Central Hospital, Guangdong Medical University, Dongguan, China

**Keywords:** manganese, preeclampsia, gut microbiota, metabolic pathways, placenta

## Abstract

**Background:**

Preeclampsia (PE), a leading cause of maternal and fetal morbidity, remains poorly understood mechanistically. While metal elements like manganese (Mn) are critical for placental function, their interplay with gut microbiota in PE pathogenesis is underexplored. This study evaluates placental heavy metal exposure—particularly Mn—and its interaction with gut microbiota in modulating PE risk.

**Methods:**

The study included 21 healthy pregnant women (Control group), and 21 pregnant women diagnosed with PE (PE group). Placental samples were collected to measure metal elements concentrations, while fecal samples were obtained to assess gut microbiota composition. Associations between gut microbiota, PE, and placental Mn levels were analyzed using the Analysis of Composition of Microbiomes with Bias Correction 2 method. Additionally, KEGG pathway enrichment analysis was conducted to identify metabolic pathways linked to PE and Mn levels.

**Results:**

Mn levels were significantly lower in the PE group compared to the Control group (*p* = 0.002). Gut microbiota diversity showed no significant differences between groups, but specific genera were linked to PE and Mn levels: *Campylobacter* and *Porphyromonas* were positively correlated with PE and negatively with Mn, while *Coprobacillus* showed the opposite pattern. KEGG pathway enrichment analysis identified eight metabolic pathways negatively associated with PE and positively linked to Mn, including the degradation of aromatic compounds.

**Conclusion:**

Our findings suggest that Mn may serve as a protective factor against PE within a certain concentration range. Interactions between Mn and specific bacterial genera (*Coprobacillus*, *Campylobacter*, and *Porphyromonas*) appear to influence PE development by altering gut microbiota metabolic activities. These findings underscore the potential significance of the gut microbiota-Mn interplay in PE pathogenesis.

## Introduction

Preeclampsia (PE) is a multisystem pregnancy disorder involving varying degrees of placental malperfusion, which leads to the release of soluble factors into the maternal circulation. These factors cause maternal vascular endothelial injury, leading to hypertension and damage to multiple organs (Chiang et al., 2024; Chappell et al., 2021). As a major contributor to maternal and perinatal morbidity and mortality, PE imposes a significant global health burden. The global incidence of PE is estimated to be 4.5%, which is similar to its incidence in China (Abalos et al., 2013; Li et al., 2021). PE is associated with adverse fetal outcomes, such as fetal growth restriction (FGR), preterm delivery, and stillbirth (Li et al., 2021; Harmon et al., 2015; Poon et al., 2019). It also has long-term health consequences for both pregnant women and their offspring, including cardiovascular disease, diabetes, dyslipidemia, and, in children, attention-deficit/hyperactivity disorder (ADHD) (Pittara et al., 2021). Due to its serious consequences, preeclampsia has been extensively studied. Research has identified maternal vascular malperfusion as a key factor in its pathogenesis (Ernst, 2018; Khong et al., 2016). However, the upstream causes remain unclear.

Recent studies have focused on the association between preeclampsia and metal elements, especially manganese (Mn). Mn is an essential trace element involved in enzyme synthesis, activation, and the regulation of glucose and lipid metabolism (Li and Yang, 2018). Most studies have reported a negative correlation between Mn levels and preeclampsia, though the underlying mechanisms remain unclear (Chen et al., 2024; Liu et al., 2019; Liu et al., 2020; Borghese et al., 2023; Enebe et al., 2023). One possible explanation is that Mn is a key component of superoxide dismutase (SOD), an enzyme that neutralizes reactive oxygen species (e.g., superoxide anions) linked to hypertension, thus reducing preeclampsia risk (Li and Yang, 2018; Schlichte et al., 2021). Other metal elements, including Nickel (Ni), copper (Cu), arsenic (As), cadmium (Cd), and lead (Pb), have also been reported to be associated with PE (He et al., 2024). Notably, most current studies primarily focus on detecting metal elements in serum, with only a few examining their levels in the placenta. Therefore, one of the objectives of this study is to explore the association between metal elements, particularly Mn in the placenta, and preeclampsia, as well as their underlying mechanisms.

In addition to manganese, the gut microbiota is also considered closely linked to preeclampsia. The gut microbiota plays a vital role in human metabolism and immune regulation and is strongly associated with the development of diseases such as preeclampsia (Zambella et al., 2024; Zong et al., 2023; Deady et al., 2024). A 2021 study revealed that preeclamptic patients exhibited significantly lower relative abundances of Var*ibaculum*, *Prevotella*, *Lactobacillus*, and *Porphyromonas* in their gut microbiota compared to healthy pregnant women. These bacteria can produce short-chain fatty acids (SCFAs), such as butyrate and propionate. These metabolites support intestinal barrier integrity and help modulate the immune system (Huang et al., 2021). Similarly, another study highlighted *Limosilactobacillus Fermentum* as a key bacterium linked to severe preeclampsia (Liu et al., 2024). It may mitigate severe preeclampsia by enhancing arginine and proline metabolism and influencing the flagellar assembly functions of the gut microbiota. However, most studies on the gut microbiota and preeclampsia emphasize bacterial relative abundances, with research on absolute abundances remaining limited (Huang et al., 2021; Wang et al., 2020; Zhao et al., 2023; Meijer et al., 2023; Chang et al., 2020). Furthermore, these studies frequently employ Linear Discriminant Analysis Effect Size (LefSe), a method sensitive to sparse data and unable to adjust for confounding factors like age and body mass index (BMI). Thus, more robust analytical methods are crucial for investigating gut microbiota differences between preeclamptic and healthy pregnant women.

Although emerging evidence above suggests that both the gut microbiota and trace elements contribute to the pathogenesis of preeclampsia, their intricate interplay remains unclear. Current studies have revealed that metal exposure, such as manganese, can affect gut microbiota composition, potentially promoting or preventing the development of certain diseases (Zhu et al., 2024). However, human studies on this topic remain limited, especially in vulnerable groups like pregnant women. Therefore, this study compares gut microbiota and metal element levels between healthy pregnant women and those with preeclampsia. Particular attention is given to the relationship among preeclampsia, gut microbiota, and placental manganese levels. Our findings may provide new insights into the mechanisms underlying preeclampsia.

## Methods

### Participants

Participants were recruited from Dongguan Songshan Lake Central Hospital, Guangdong Province, China, between November 2022 and October 2023. Eligible participants were pregnant women aged 18 years or older, with a gestational age of more than 32 weeks, residing in Dongguan for over 1 year, and willing to provide informed consent.

The exclusion criteria were as follows: (1) A history of preexisting conditions such as hypertension, diabetes mellitus, coronary heart disease, cerebral infarction, kidney disease, cancer, glucose-6-phosphate dehydrogenase deficiency, or other metabolic or immune disorders; (2) A history of acute gastroenteritis, bacterial urinary tract infections, inflammatory bowel disease, or other chronic conditions that may affect gut microbiota; (3) A family history of hypertension (including gestational hypertension), coronary artery disease, diabetes mellitus, or kidney disease; (4) Use of mineral supplements, antibiotics, probiotics, prebiotics, proton pump inhibitors (PPIs), or other medications that may influence body metal element levels or gut microbiota after 28 weeks of gestation; (5) Twin or multiple pregnancies, assisted reproduction, severe memory impairment, mental or neurological disorders, or a history of seizures or loss of consciousness.

A total of 42 pregnant women were included in the study: 21 were healthy, and 21 were diagnosed with preeclampsia based on the Diagnosis and Treatment of Hypertension and Preeclampsia in Pregnancy: A Clinical Practice Guideline in China (2020) (Hypertensive Disorders in Pregnancy Subgroup, Chinese Society of Obstetrics and Gynecology, Chinese Medical Association, 2020).

Written informed consent was obtained from all participants. To ensure privacy, personal identifiers were replaced with codes consisting of the participant’s initials and admission numbers during chemical and microbiological analyses. For statistical analyses, only these codes were used, with all personal information excluded. The study protocol was approved by the Ethics Committee of Dongguan Songshan Lake Central Hospital.

### Data collection

Demographic data, including age, height, pre-pregnancy weight, pre-pregnancy body mass index (BMI), delivery weight, and delivery BMI, as well as clinical data such as biochemical markers [e.g., Total Protein (TP) and Aspartate Aminotransferase (AST)] and hematological markers [e.g., White Blood Cell Count (WBC) and Red Blood Cell Count (RBC)], were obtained from hospital medical records. For statistical analysis, delivery BMI was used in place of pre-pregnancy BMI. Similarly, clinical measurements closest to the time of delivery were selected to ensure temporal consistency, as placental samples were collected postpartum.

Placental tissues were collected immediately following delivery. To ensure sample integrity, peripheral margins, necrotic areas, and calcified regions were excluded. After thorough rinsing with sterile saline, two tissue samples—each approximately 2 cm in diameter—were excised from the central and peripheral regions of each placenta. The final metal concentration for each placenta was calculated as the average of these two samples. All specimens were immediately flash-frozen in liquid nitrogen and stored at −80 °C until analysis. For metal extraction, tissues were subjected to microwave-assisted acid digestion using nitric acid (HNO₃) and hydrogen peroxide (H₂O₂) to fully decompose organic material and release metal ions into solution. Metal concentrations, including manganese (Mn), nickel (Ni), copper (Cu), arsenic (As), cadmium (Cd), and lead (Pb), were subsequently quantified using liquid chromatography–inductively coupled plasma mass spectrometry (LC-ICP-MS). Results were reported in micrograms per gram (μg/g) of tissue.

Approximately 5 grams of fresh stool were collected from each participant within 1 week prior to their estimated due date and placed into sterile collection tubes. The samples were transported to the laboratory within 2 h and immediately stored at −80 °C for subsequent analysis. Absolute quantitative 16S rRNA gene sequencing was performed using the Accu16S method, which incorporates synthetic DNA sequences as internal standards for precise quantification (Genesky Biotechnologies Inc, n.d.). Details of the instruments and reagents used for gene sequencing are listed in Supplementary Tables S1, S2. All microbial analyses in this study were conducted based on absolute copy numbers.

### Statistical analysis

Demographic data, clinical data, and placental metal data are expressed as mean (standard deviation). Group differences were assessed using the student *t*-test for data meeting normality and homogeneity of variance. For data that violated these assumptions, the Mann–Whitney U test was applied. Normality was evaluated using the Shapiro–Wilk test, and homogeneity of variance was assessed with the Levene test.

Group differences in α-diversity were analyzed using linear regression if the data met the normality assumption, or a Generalized Linear Model (GLM) if the data did not meet the normality assumption. *p*-values were adjusted for multiple comparisons using the Bonferroni correction (False Discovery Rate, FDR) to assess the significance of species diversity. Differences in β-diversity were evaluated using Principal Coordinates Analysis (PCoA). Both α-diversity and β-diversity analyses were adjusted for age and delivery BMI.

Gut microbiota analyses were performed at the genus level. Associations between bacterial genera and preeclampsia or placental metal elements were evaluated using Analysis of Composition of Microbiomes with Bias Correction 2 (ANCOM-BC2) (Lin and Peddada, 2024), with *p*-values adjusted by the Benjamini-Hochberg method. Differential KEGG (Kyoto Encyclopedia of Genes and Genomes) Orthology genes were identified through the KEGG database (Kanehisa and Goto, 2000), and pathway enrichment analysis was conducted to highlight significant changes in metabolic pathways. Particular attention was given to bacterial genera, KEGG Orthology (KO) genes, and metabolic pathways significantly associated with both preeclampsia and placental manganese levels.

Statistical analyses were performed in R (version 4.4.2). Visualizations were generated using R (version 4.4.2) and Photoshop (version 26.2). All tests were two-tailed, with a *p*-value < 0.05 considered statistically significant.

## Results

### Demographic and clinical data

A total of 42 pregnant women were included in the study, with 21 healthy pregnant women serving as the control group and 21 women diagnosed with preeclampsia (PE) comprising the PE group. Their demographic and clinical data are presented in Table 1. The mean age was comparable between the control group (28.90 ± 5.64 years) and the PE group (29.95 ± 5.26 years, *p* = 0.524). Similarly, no significant differences were observed in height, weight or BMI (*p* > 0.05). Regarding liver function indicators, the PE group exhibited significantly elevated aspartate aminotransferase (AST) levels compared to the control group (28.87 ± 19.87 U/L vs. 20.70 ± 2.97 U/L, *p* = 0.018). However, alanine aminotransferase (ALT) and the AST/ALT ratio did not differ significantly between the groups. In terms of hematological parameters, there was a significant increase in lymphocyte ratio (LYMPH) in the PE group compared to the control group (0.27 ± 0.26 vs. 0.19 ± 0.11, *p* = 0.024), whereas other indices, including white blood cell count (WBC), red blood cell count (RBC), hematocrit (HCT), and neutrophil ratio (NEUT), showed no statistically significant differences (*p* > 0.05).

**Table 1:** Comparison of demographic and clinical data between the control group and the PE group.

Characteristics	Control (*n* = 21)	PE (*n* = 21)	*p* value
Age (years)	28.90 (5.64)	29.95 (5.26)	0.524
Height (cm)	158.38 (4.49)	159.71 (6.08)	0.209
Early pregnancy weight (kg)	56.04 (12.21)	60.41 (13.11)	0.249
Early pregnancy BMI (kg∙m^−2^)	66.92 (14.36)	70.41 (13.77)	0.146
Delivery weight (kg)	22.24 (4.28)	23.64 (4.76)	0.323
Delivery BMI (kg∙m^−2^)	26.52 (5.05)	27.63 (4.88)	0.283
TP (g/L)	57.86 (24.54)	62.51 (6.37)	0.151
GLO (g/L)	26.19 (11.15)	28.78 (3.63)	0.606
Alb (g/L)	31.70 (13.50)	33.66 (3.49)	0.059
AST (U/L)	20.70 (2.97)	28.87 (19.87)	0.018
ALT (U/L)	16.16 (3.78)	27.74 (45.45)	0.800
AST/ALT	1.38 (0.24)	1.35 (0.38)	0.757
WBC (10^9^/L)	9.55 (2.09)	17.38 (19.07)	0.941
RBC (10^12^/L)	4.11 (0.42)	5.52 (3.00)	0.358
HCT	0.36 (0.04)	1.49 (5.23)	0.378
NEUT	0.75 (0.05)	0.91 (0.91)	0.222
LYMPH	0.19 (0.11)	0.27 (0.26)	0.024

PE, Preeclampsia; BMI, Body Mass Index; TP, Total Protein; GLO, Globulin; Alb, Albumin; AST, Aspartate Aminotransferase; ALT, Alanine Aminotransferase; AST/ALT, Ratio of Aspartate to Alanine Aminotransferase; WBC, White Blood Cell Count; RBC, Red Blood Cell Count; HCT, Hematocrit; NEUT, Neutrophil Ratio; LYMPH, Lymphocyte Ratio.

### Placental metal element levels

**Figure 1:**
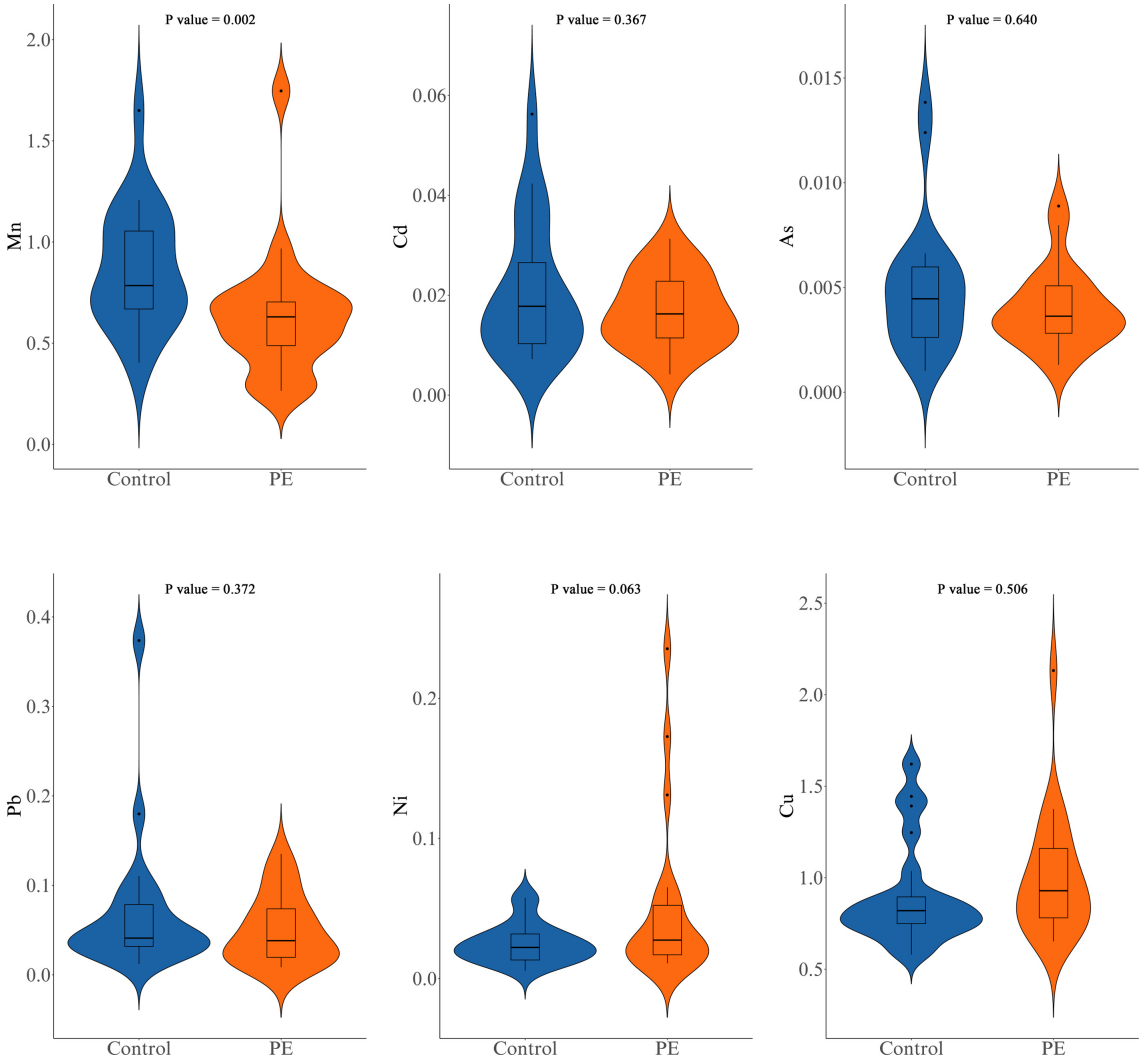
Comparison of manganese (Mn), cadmium (Cd), arsenic (As), lead (Pb), nickel (Ni), and copper (Cu) levels between the control group and the PE group.

The levels of various metal elements were compared between healthy pregnant women and women with preeclampsia (Figure 1; Supplementary Table S3). Mn levels were significantly lower in the PE group than in the Control group (0.60 ± 0.31 μg/g vs. 0.86 ± 0.30 μg/g, *p* = 0.002). No significant differences were found for Ni (*p* = 0.063), Cu (*p* = 0.506), As (*p* = 0.640), Cd (*p* = 0.367), or Pb (*p* = 0.372).

### Gut microbiota diversity analysis

**Figure 2:**
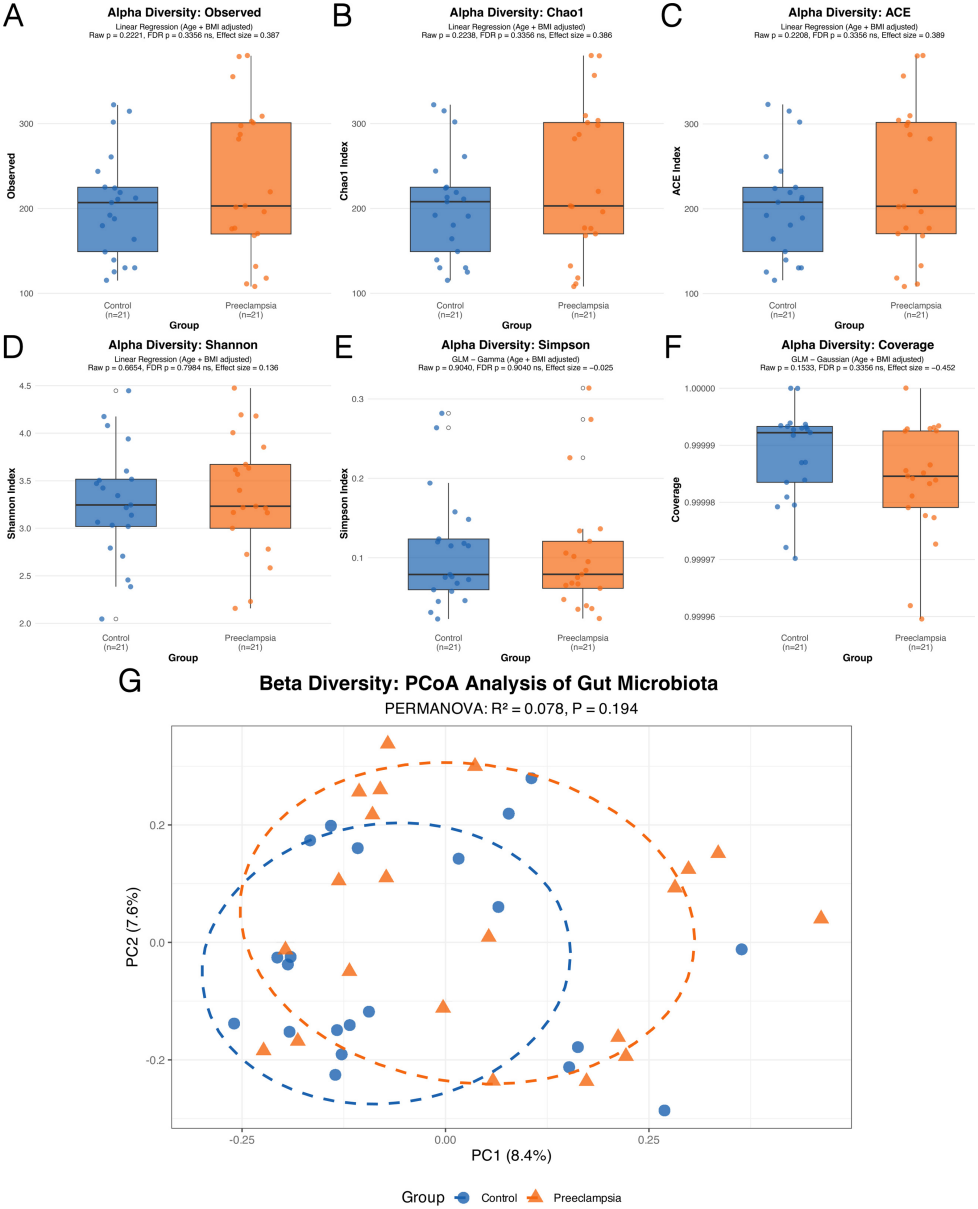
Comparison of gut microbiota α-diversity **(A–F)** and β-diversity **(G)** between the Control group and the PE group.

The gut microbiota diversity between the two groups was compared in terms of both α- and β-diversity. For α-diversity (Figures 2A–F), the following indices were analyzed: Observed Species (the count of distinct species observed), Chao 1 Estimator and Abundance-based Coverage Estimator (both estimators of species richness that account for unobserved species), Shannon Diversity Index and Simpson’s Diversity Index (which measure both diversity and evenness within a community), and Coverage (estimating the proportion of the total species that are represented in the sample). None of these indices showed statistically significant differences between the two groups (all *p* > 0.05), suggesting no significant differences in α-diversity. For β-diversity (Figure 2G), PCoA demonstrated no clear separation between the groups (*p* = 0.194), with the first principal coordinate accounting for 8.4% of the variance and the second 7.6%. These results suggest that β-diversity may also lack significant differences between healthy pregnant women and those with preeclampsia.

### Differential bacteria at the genus level

**Figure 3:**
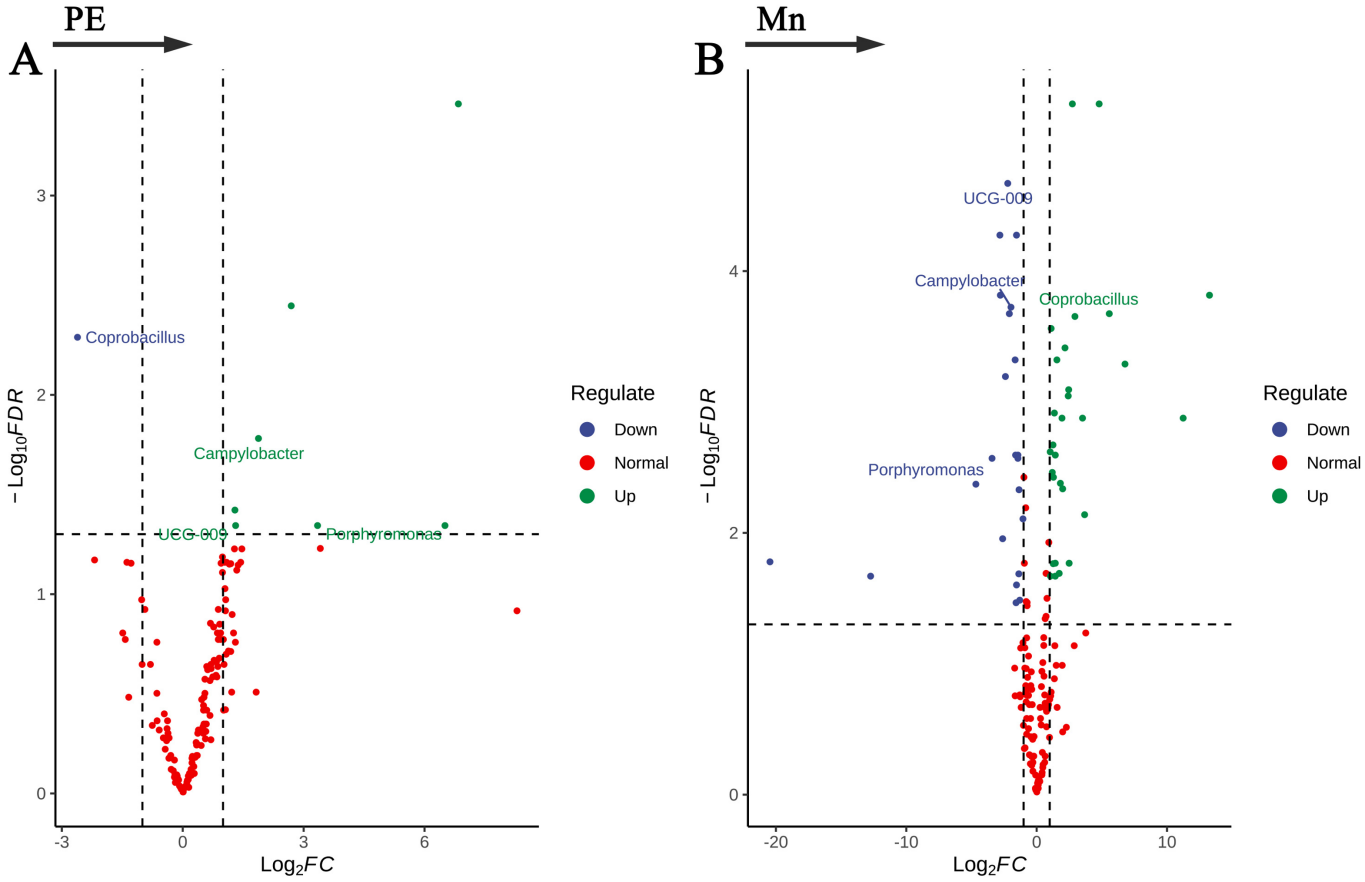
Volcano plots of bacterial genera significantly associated with **(A)** preeclampsia and **(B)** placental manganese levels.

In this study, we investigated the relationships between the gut microbiota, preeclampsia, and placental Mn levels at the genus level, employing the ANCOM-BC2 analytical method, which was adjusted for age and delivery BMI. The results are shown in Figure 3 and Supplementary Table S4. Our findings highlighted distinct microbial compositions associated with PE and placental Mn levels. Four bacterial genera were identified as having significant correlations with PE and Mn levels. *Campylobacter*, *Porphyromonas*, and *UCG-009* showed positive associations with PE but negative associations with Mn levels. Conversely, *Coprobacillus* was negatively correlated with PE and positively correlated with Mn levels. The specific log2 fold changes (log2FC) and FDR underscore the robustness and significance of these associations. These results indicate specific dysbiosis patterns in the gut microbiota related to PE and Mn.

### KEGG pathway enrichment analysis

**Figure 4:**
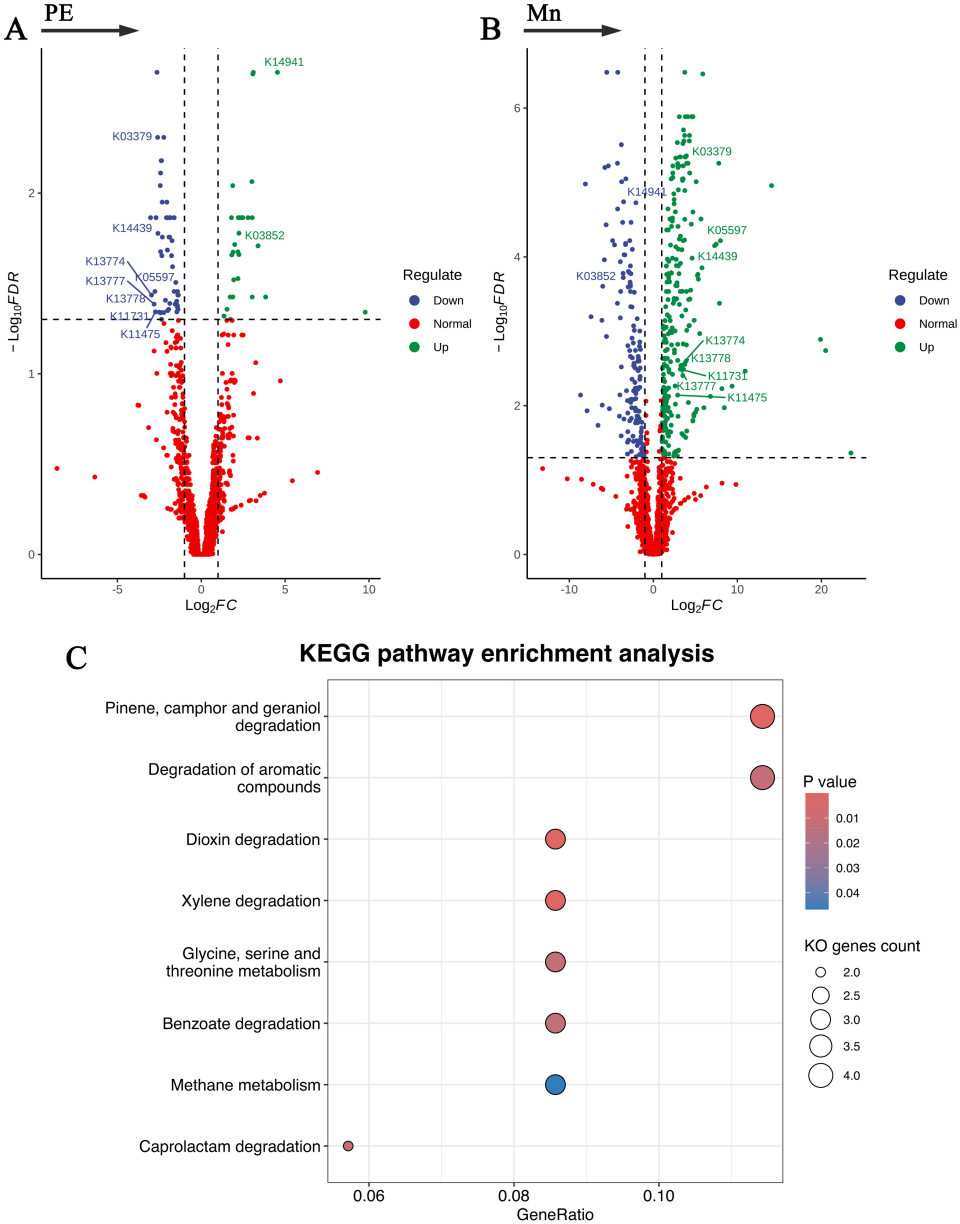
Functional capacities of the gut microbiota in relation to preeclampsia and placental manganese levels. **(A,B)** Volcano plots of KO genes significantly associated with preeclampsia and placental manganese levels. **(C)** Bubble plot of KEGG pathway enrichment analysis for the selected KO genes.

The potential functional capacities of the gut microbiota in relation to preeclampsia and placental manganese levels were explored by mapping microbial genes to the KEGG databases, with the findings detailed in Figure 4 and Supplementary Table S5. We identified 81 distinct KEGG Orthology (KO) genes: 29 exhibited a positive correlation with preeclampsia but a negative correlation with Mn; conversely, 52 showed a negative correlation with preeclampsia and a positive correlation with Mn. Subsequent KEGG pathway enrichment analysis of these KO genes revealed eight metabolic pathways negatively associated with preeclampsia yet positively associated with Mn. These pathways include: Pinene, camphor, and geraniol degradation; Dioxin degradation; Xylene degradation; Caprolactam degradation; Degradation of aromatic compounds; Glycine, serine, and threonine metabolism; Benzoate degradation; Methane metabolism. These results underscore metabolic alterations within the gut microbiota in response to fluctuations in both preeclampsia and manganese levels. These findings lay a foundation for further investigation into the role of microbial genes in influencing host metabolic processes, thereby elucidating the complex pathophysiology of preeclampsia associated with manganese.

## Discussion

This study investigates the relationship between placental trace element levels, specifically manganese, gut microbiota, and preeclampsia. We identified a negative correlation between placental Mn levels and preeclampsia. Additionally, our findings suggest that interactions between Mn and four bacterial genera—*Coprobacillus*, *Campylobacter*, *Porphyromonas*, and *UCG-009*—may influence the development of preeclampsia. This effect may be linked to altered gut microbiota metabolic activities, including the degradation of exogenous organic pollutants and the metabolism of methane, purines, and amino acids.

In this study, six metal elements were measured: Mn, Ni, Cu, As, Cd, and Pb. Among them, Mn is distinct. Previous studies have generally reported positive or null associations between the other metals and preeclampsia (Chen et al., 2024; Borghese et al., 2023; Hernandez-Castro et al., 2024; Sandoval-Carrillo et al., 2016), whereas Mn has been widely recognized as a protective factor. A recent meta-analysis (Wu et al., 2024), encompassing 18 observational studies, evaluated the relationship between maternal Mn levels and PE. The analysis revealed that lower serum Mn levels were associated with a higher risk of PE, regardless of the geographic region or the timing of serum Mn collection. Our study similarly supports this association. However, we chose to analyze placental Mn levels rather than serum levels. Although the protective role of Mn in PE is supported by a growing body of evidence, the underlying mechanisms remain unclear. One widely accepted hypothesis suggests that Mn contributes to the activity of manganese superoxide dismutase (Mn-SOD), a critical antioxidant enzyme. Mn-SOD mitigates oxidative stress by scavenging reactive oxygen species (ROS), thereby alleviating inflammation and reducing placental damage (Schlichte et al., 2021; Jahan et al., 2023; Grzeszczak et al., 2023; Tossetta et al., 2023; Liu et al., 2022; Matsubara et al., 2015). Additionally, ROS may impair blood pressure regulation by promoting endothelial dysfunction (Silva et al., 2012), while Mn-SOD can alleviate preeclampsia by counteracting this effect (Glover et al., 2014; Tomimatsu et al., 2019; Gong et al., 2020).

Besides Mn-SOD, our findings suggest that manganese may indirectly mitigate the progression of preeclampsia by altering the composition of the gut microbiota. Increasing evidence highlights that exposure to metal elements, such as Mn, can influence gut microbiota composition (Li et al., 2021; Peng et al., 2024). However, most studies have been conducted in animal models. Research on the relationship between Mn and the gut microbiota in humans—especially in pregnant women—remains limited. Our study identified associations between Mn and 63 bacterial genera, four of which—*Coprobacillus*, *Campylobacter*, *Porphyromonas*, and *UCG-009*—were also associated with preeclampsia. In particular, “UCG-009” refers to an unclassified genus group with minimal research available. Therefore, in this paragraph, let us focus on the other three genera. *Campylobacter* and *Porphyromonas* are common human pathogens. In our study, both genera were positively associated with preeclampsia and negatively correlated with placental manganese (Mn) levels. *Campylobacter* is a well-established cause of gastroenteritis and has been linked to Guillain-Barré syndrome (Butzler, 2004; Nachamkin et al., 1998). Similarly, *Porphyromonas* is associated with periodontal diseases such as periodontitis and is considered a risk factor for adverse pregnancy outcomes, including preeclampsia (Slots, 1999; Chopra et al., 2020). A shared characteristic of the two pathogens is their capacity to provoke inflammatory responses, resulting in elevated levels of pro-inflammatory cytokines in the body (Zhang et al., 2022; Young et al., 2007; Mukherjee et al., 2024; Kang et al., 2022). This pro-inflammatory activity may underlie their association with preeclampsia, highlighting the potential role of inflammatory mechanisms in the disease’s pathogenesis. In contrast, *Coprobacillus* demonstrated a positive correlation with placental Mn levels and a negative association with preeclampsia. Previous studies suggest that this genus produces SCFAs, which are known to benefit human health and can reduce inflammation (Yang et al., 2024; Yu et al., 2021; Hu et al., 2023; Liu et al., 2022).

The three genera—*Coprobacillus*, *Campylobacter*, and *Porphyromonas*—highlighted in our study have been rarely reported in prior research. In another study conducted in Liaocheng, China, *Akkermansia muciniphila*, a bacterium known for producing SCFAs, was found to be reduced in PE patients. Animal experiments further showed that supplementation with *Akkermansia muciniphila*, propionic acid, or butyric acid alleviated several features of preeclampsia in rats (Jin et al., 2022). Similarly, a study in Guangzhou, China, found that not only was *Akkermansia* reduced in PE patients, but another beneficial bacterium, *Faecalibacterium*—also known for butyrate production—was diminished as well (Chen et al., 2020). An Australian study also reported a reduction in the butyrate-producing genus *Coprococcus* among PE patients (Altemani et al., 2021). However, our study did not detect significant differences in the abundance of *Akkermansia, Faecalibacterium*, or *Coprococcus*. These discrepancies could stem from variations in the timing of fecal sample collection or differences in microbial composition analysis methods. Despite these inconsistencies, both our findings and prior research consistently underscore a reduction in SCFA-producing, inflammation-regulating beneficial bacteria in PE patients. This supports the hypothesis that SCFAs may exert a protective effect against PE (Mackay and Marques, 2022; Cui et al., 2023).

KEGG metabolic pathway prediction based on Accu16S data suggested eight potentially downregulated pathways in patients with PE. Six of these predicted pathways—dioxin degradation, xylene degradation, caprolactam degradation, benzoate degradation, pinene/camphor/geraniol degradation, and aromatic compound degradation—are related to microbial xenobiotic metabolism and may be involved in the degradation of exogenous organic pollutants. Such pollutants have been reported to activate the aryl hydrocarbon receptor (AHR), thereby contributing to inflammation and oxidative stress (Vogel et al., 2020). The other two predicted pathways—glycine, serine, and threonine metabolism, and methane metabolism—also have potential links to inflammation and oxidative stress (Ye et al., 2020; Aguayo-Cerón et al., 2023; Egbujor et al., 2024). Nevertheless, it should be emphasized that these findings are based on predictive functional profiling of microbial communities rather than direct metabolomic measurements. Direct evidence linking these pathways or metabolites to preeclampsia remains limited. Further validation, particularly through metabolomic analyses and mechanistic studies in animal models, is warranted to clarify these potential associations.

The primary strength of this study is its comprehensive approach, which adopts a tripartite perspective to investigate the interplay among placental manganese levels, gut microbiota composition, and preeclampsia. Rather than focusing on a single factor, such as gut microbiota or manganese, our study integrates these factors to provide novel insights into the potential mechanisms by which manganese may mitigate the risk of preeclampsia through modulating gut microbiota composition. Furthermore, we employed ANCOM-BC2, a robust analytical method published in *Nature Methods* in 2024 (Lin and Peddada, 2024), to precisely evaluate differences in gut microbiota composition. Despite these strengths, our study has several limitations. First, the sample size was relatively small, with only 21 pregnant women in the PE group and 21 in the control group, which may limit statistical power. Second, placenta and fecal samples were collected at delivery or within 1 week before delivery, when preeclampsia was already established. Therefore, the observed microbial and metabolic alterations may represent consequences of the disease rather than predisposing risk factors, limiting causal interpretation. To address these issues, future studies should adopt larger, multi-center cohorts with longitudinal sampling starting early in pregnancy to validate our findings and clarify temporal and causal relationships.

## Data Availability

The datasets presented in this study can be found in online repositories. The names of the repository/repositories and accession number(s) can be found at: https://db.cngb.org/data_resources/?query=CNP0006841, China National GeneBank DataBase (CNGBdb), accession code CNP0006841.
