# Supplementary material for: Association of placental manganese levels, maternal gut microbiota, and preeclampsia: a tripartite perspective
Source: Front Microbiol. 2025 Oct 20;16:1674549. doi: 10.3389/fmicb.2025.1674549 (PMC12580270; doi:10.3389/fmicb.2025.1674549)
Supplement: Supplementary file 1 [file Data_Sheet_1.PDF]

Supplements

Title: Association of Placental Manganese Level, Maternal Gut Microbiota, and Preeclampsia: A Tripartite Perspective

First author: Tianze Ding

Table S1. Laboratory Apparatuses Used in Gene Sequencing

Table S2. Laboratory Reagents Used in Gene Sequencing

Table S3. Comparison of Metal Elements Levels Between the Control Group and the PE Group

Table S4. The Four Bacterial Genera Selected by ANCOM-BC2 and Their Associations with Preeclampsia and Placental Manganese Levels

Table S5. Results of KEGG Metabolic Pathway Enrichment Analysis

This supplementary material has been provided by the authors to give readers additional information about their work.

Table S1. Laboratory Apparatuses Used in Gene Sequencing

Apparatuses	Suppliers
Qubit 3.0 Fluorometer	Thermo Fisher Scientific, USA
Invitrogen Qubit3.0 Spectrophotometer	Thermo Fisher Scientific, USA
Vortex Genius 3	IKA, Germany
Agilent 2100 bioanalyzer	Agilent Technologies, USA
2.5/10/200/1000μl pipettor	Eppendorf, Germany
10/200/1000μl Pipette Tips	Axygen
0.2 mL PCR tubes, 1.5 mL centrifuge tubes, and 96-well plates	Axygen
ABI 2720 Thermal Cycler	Thermo Fisher Scientific, USA
Eppendorf 5810R Centrifuge	Eppendorf, Hamburg, Germany
Illumina NovaSeq	Illumina, San Diego, CA, USA

Table S2. Laboratory Reagents Used in Gene Sequencing

Reagents	Suppliers
VAHTS DNA Clean Beads	Vazyme, China
TransStart TopTaqDNA Polymerase kit	Transgen, China
HIDI	Thermo Fisher Scientific, USA
dNTP mix (2.5mM)	Takara, China
5× Seq Buffer	Takara, China
SNaPshot Mix	Thermo Fisher Scientific, USA
rSAP	Thermo Fisher Scientific, USA
GeneScan-LIZ120	Thermo Fisher Scientific, USA
GeneScan-LIZ500	Thermo Fisher Scientific, USA
Takara HotStart Taq (5U/μL)	Takara
NovaSeq Reagent kit	Illumina, USA

Table S3. Comparison of Metal Elements Levels Between the Control Group and the PE Group

Metal elements	Control (n = 21)	PE (n = 21)	P value
Mn (μg/g)	0.86 (0.30)	0.60 (0.31)	0.002
Ni (μg/g)	0.03 (0.01)	0.05 (0.06)	0.063
Cu (μg/g)	0.94 (0.29)	1.00 (0.33)	0.506
As (μg/g)	0.00 (0.00)	0.00 (0.00)	0.640
Cd (μg/g)	0.02 (0.01)	0.02 (0.01)	0.367
Pb (μg/g)	0.07 (0.08)	0.06 (0.04)	0.372

Abbreviations: PE, Preeclampsia; Mn, Manganese; Ni, Nickel; Cu, Copper; As, Arsenic; Cd, Cadmium; Pb, Lead.

Table S4. The Four Bacterial Genera Selected by ANCOM-BC2 and Their Associations with Preeclampsia and Placental Manganese Levels

Genera	PE		Mn	
	log2FC	FDR	log2FC	FDR
Coprobacillus	-2.613	0.005	2.932	0.000
Campylobacter	1.878	0.017	-1.969	0.000
Porphyromonas	3.345	0.045	-3.432	0.003
UCG-009	1.313	0.045	-2.231	0.000

Abbreviations: PE, Preeclampsia; Mn, Manganese; log2FC, log2 Fold Changes; FDR, False Discovery Rates.

Table S5. Results of KEGG Metabolic Pathway Enrichment Analysis

ID	Category	Description	P_adjust	geneID
ko00907	Metabolism of terpenoids and polyketides	Pinene, camphor and geraniol degradation	0.000	K11731<br>K13774<br>K13777<br>K13778
ko00621	Xenobiotics biodegradation and metabolism	Dioxin degradation	0.001	K18364<br>K18365<br>K18366
ko00622	Xenobiotics biodegradation and metabolism	Xylene degradation	0.001	K18364<br>K18365<br>K18366
ko00930	Xenobiotics biodegradation and metabolism	Caprolactam degradation	0.010	K00496<br>K03379
ko01220	Global and overview maps	Degradation of aromatic compounds	0.011	K03379<br>K18364<br>K18365<br>K18366
ko00260	Amino acid metabolism	Glycine, serine and threonine metabolism	0.012	K00304<br>K00305<br>K00479
ko00362	Xenobiotics biodegradation and metabolism	Benzoate degradation	0.012	K18364<br>K18365<br>K18366
ko00680	Energy metabolism	Methane metabolism	0.047	K00126<br>K15228<br>K15229

All pathways were negatively correlated with preeclampsia and positively correlated with placental manganese levels.
